# Adherence to low carbohydrate diet and prevalence of psychological disorders in adults

**DOI:** 10.1186/s12937-019-0513-8

**Published:** 2019-12-23

**Authors:** Soraiya Ebrahimpour-Koujan, Ammar Hassanzadeh Keshteli, Hamid Afshar, Ahmad Esmaillzadeh, Peyman Adibi

**Affiliations:** 1 Students’ Scientific Research Center, Tehran University of Medical Sciences, Tehran, Iran; 2 Department of Community Nutrition, School of Nutritional Sciences and Dietetics, Tehran University of Medical Sciences, PO Box 14155-6117, Tehran, Iran; 3 Department of Medicine, University of Alberta, Edmonton, Alberta Canada; 4 Integrative Functional Gastroenterology Research Center, Isfahan University of Medical Sciences, Isfahan, Iran; 5 Psychosomatic Research Center, Isfahan University of Medical Sciences, Isfahan, Iran; 6 Obesity and Eating Habits Research Center, Endocrinology and Metabolism Molecular -Cellular Sciences Institute, Tehran University of Medical Sciences, Tehran, Iran; 7 Department of Community Nutrition, School of Nutrition and Food Science, Isfahan University of Medical Sciences, Isfahan, Iran

**Keywords:** Low carbohydrate diet, Psychological disorders, FFQ, Cross-sectional

## Abstract

**Background:**

Although individual macronutrients were studied in relation to mental health, no information exist about the association between adherence to low carbohydrate diet and psychological disorders. This study was conducted to investigate the association between adherence to a low carbohydrate diet and prevalence of psychological disorders among Iranian adults.

**Methods:**

In this cross-sectional study on 3362 adult men and women, dietary intakes were examined by the use of a validated semi-quantitative food frequency questionnaire. Low carbohydrate diet (LCD) score was computed for each participant based on deciles of percentages of energy from macronutrients. Then the scores of carbohydrate, protein and fat intake for each participant were summed up to achieve the overall LCD score, which ranged from 3 (highest carbohydrate intake and lowest fat and protein intakes) to 30 (lowest carbohydrate intake and highest fat and protein intakes). Anxiety, depression, and psychological distress were assessed by validated Iranian versions of the Hospital Anxiety and Depression Scale and General Health Questionnaire-12.

**Results:**

Prevalence of depression, anxiety and psychological distress in the whole population were 28.0, 13.3 and 22.6%, respectively. No significant differences were observed in the distribution of depression, anxiety and psychological distress across different quartiles of LCD score. After controlling for potential confounders, no significant association was seen between LCD score and prevalence of depression (OR for the highest vs. the lowest quartile of LCD score: (1.15; 95% CI: 0.93, 1.39). Consumption of LCD was not also associated with increased risk of anxiety (0.82; 95% CI: 0.59, 1.14) and psychological distress (0.92; 95% CI: 0.72, 1.16). These associations did not alter when the analyses were done stratified by gender or BMI status.

**Conclusion:**

Adherence to the low carbohydrate diet, which contains high amount of fat and proteins but low amounts of carbohydrates, was not associated with increased odds of psychological disorders including depression, anxiety and psychological distress. Given the cross-sectional nature of the study which cannot reflect causal relationships, longitudinal studies, focusing on types of macronutrients, are required to clarify this association.

## Introduction

The common psychological disorders including depression and anxiety have considerable contribution to the global burden of disease, accounting for 7.4% of all healthy years of life lost [1]. According to global estimation of WHO, 4.4% of the general population suffer from depressive disorders, and 3.6% from anxiety disorders [2]. Among Iranian population, approximately 21% of adults are affected by mental disorders, which depression and anxiety were the most common conditions [3].

The complex interactions of social, environmental and biological factors are contributing to psychological disorders [4]. Diet has been considered as a modifiable factor that plays a key role in mental health [4]. Most research on dietary factors and psychological health has been done on micronutrients and limited information is available about macronutrients [5]. Diets low in carbohydrates and high in fats and proteins were associated with greater risk of mood disorders including anxiety and depression [6, 7]. In a prospective study, high intakes of protein were protectively associated with severe depressive symptoms [8]. Similarly, findings from a cross-sectional study revealed a positive association between low protein intakes and prevalence of mental illnesses [4]. In addition, while high dietary glycemic index was associated with increased odds of incident depression and psychological disorders [9, 10], high dietary glycemic load was inversely associated with mental disorders [10]. Low carbohydrate along with high protein and fat intakes are associated with greater satiety, which might result in a better mood [11]. It seems that the addictive effect of high carbohydrate intakes on rewards system of mid-brain by stimulating the dopamine release is the major mechanistic link between dietary carbohydrate and mental disorders [12]. High fat diet can alter the stressful behaviors through attenuating psychological problems [13]. Similarly, high protein intakes can affect the brain functioning and mental health by producing extensive ranges of neurotransmitters [14].

It must be kept in mind that most pervious findings have focused on individual macronutrients rather than their combination. A newly suggested dietary pattern, low carbohydrate diet, has considered the proportion of all macronutrients in this context. The association of this dietary pattern with metabolic diseases including metabolic syndrome, diabetes and cardiovascular disease has been examined [15–17]; however, no information is available linking this diet with psychological disorders. Examining the association of this dietary pattern with psychological disorders is particularly relevant in Middle Eastern countries where dietary carbohydrate intake composes the greatest percentage of energy intake. This study was, therefore, conducted to investigate the association between adherence to a low carbohydrate dietary pattern and psychological disorders among Iranian adults.

## Methods

### Study population

**Fig. 1:**
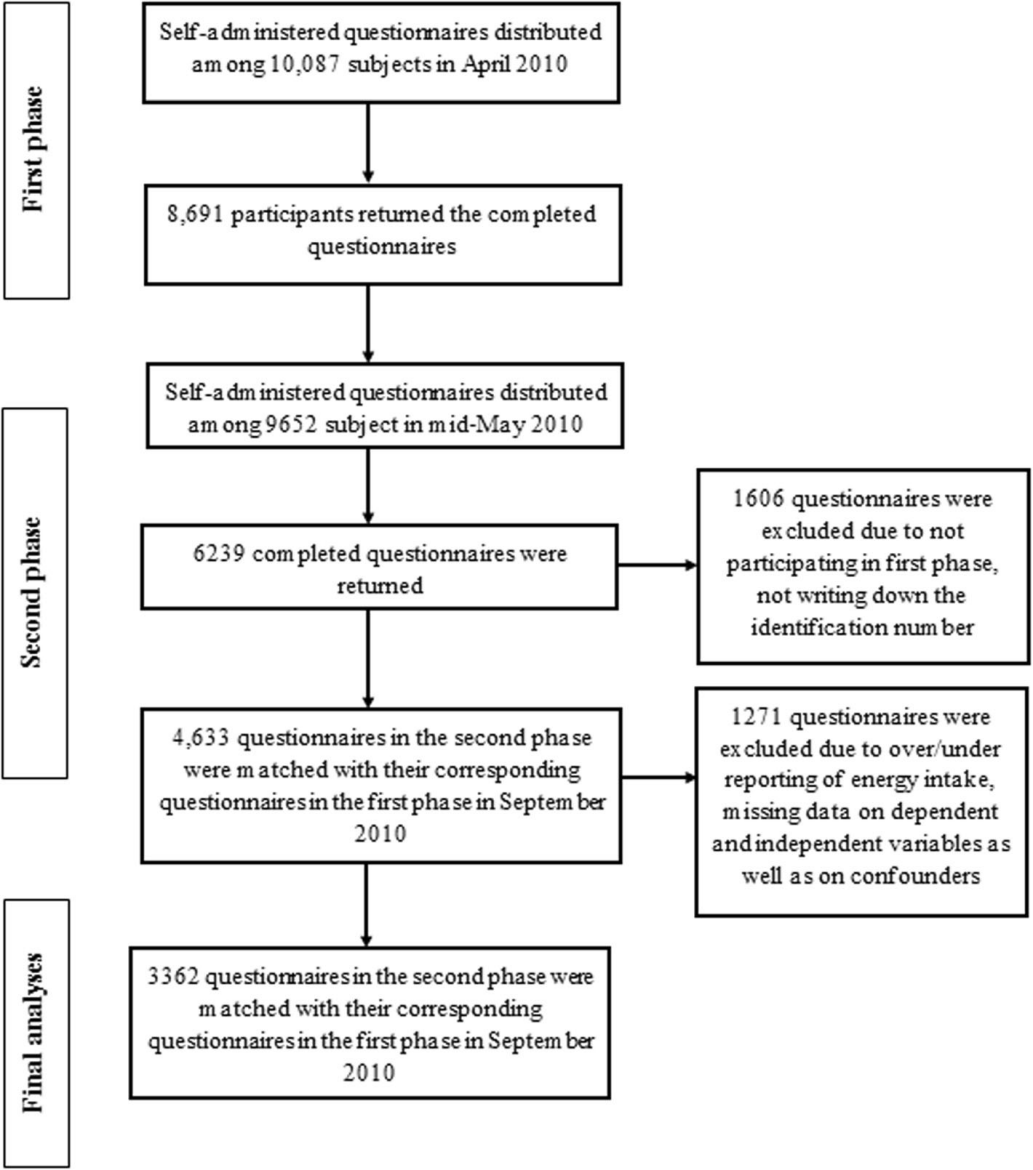
Participant’s flowchart

This cross-sectional study was performed within the “Study on the Epidemiology of Psychological, Alimentary Health and Nutrition” (SEPAHAN) project. The main aim of SEPAHAN was to investigate the association between lifestyle-related factors and functional gastrointestinal disorders. Information about study design, sampling method, data collection and participants’ characteristics are described in detail elsewhere [18]. In short, participants were recruited from the general population of Isfahan province, who were working in health centers affiliated with Isfahan University of Medical Sciences (IUMS). Data collection process of SEPAHAN project was conducted in two separate phases during April 2010 to May 2010. In the first phase, 10,087 self-administered questionnaires, containing information on demographics and dietary data, were distributed and 8691 subjects responded (response rate: 86.16%) to the questions. In the second phase, information on psychological health was collected through sending the relevant questionnaires to 9652 participants and 6239 questionnaires were completed and returned (response rate: 64.6%). No significant difference was seen in the demographic data of those returned the questionnaires and those that did not. We merged data from both phases and arrived at the sample size of 4763 people. In the current study, we excluded individuals with under- (< 800 kcal/d) and over-reporting of energy intake (> 4200 kcal/day). We also excluded individuals who had missing data on dependent and independent variables as well as on confounding factors. After these exclusions, data from 3362 participants remained for the current study. We obtained written informed consent from all subjects. The study was approved by the Regional Bioethics Committee of IUMS (no. #189069, #189082, and #189086). The flowchart of participant’s selection process is provided in Fig. 1.

### Assessment of dietary intake

Dietary intakes of daily energy, macro- and micro-nutrients were obtained using a validated Willett-format dish-based semi-quantitative food frequency questionnaire (DS-FFQ) including 106 items. This questionnaire was originally designed and validated specifically for Iranian adults [18]. Detailed information about the development of the questionnaire, its foods list and frequency response categories as well as its validity was published previously [18, 19]. The questionnaire consisted of five main categories of foods and dishes: 1) mixed dishes (cooked or canned, 29 items); 2) grains (different types of bread, cakes, biscuits and potato, 10 items); 3) dairy products (dairies, butter, and cream, 9items); 4) fruits and vegetables (22 items); and [5] miscellaneous food items and beverages (including sweets, fast foods, nuts, desserts and beverages, 36 items). Daily intakes of macro- and micro-nutrients were computed for each person using USDA food composition database modified for Iranian foods.

### Calculation of the low carbohydrate diet (LCD) score

To compute LCD score, first we categorized participants based on deciles of percentages of energy from carbohydrates, proteins, and fats. Individuals in in the lowest decile of carbohydrate intake received 10 points. Participants in second decile received 9 points and so on down to participants in the highest decile received 1 points. For fat and protein intakes, the points assigned to deciles were reversed; such that those in the highest decile received 10 points and those in the lowest decile received 1 point. We then summed up all points assigned to the three macronutrients to achieve the overall diet score, which ranged from 3 (highest carbohydrate intake and lowest fat and protein intakes) to 30 (lowest carbohydrate intake and highest fat and protein intakes). Therefore, the higher the score, the greater was the adherence to the LCD dietary pattern.

### Assessment of psychological health

The Iranian validated version of Hospital Anxiety and Depression Scale (HADS) was used to screen for anxiety and depression [19]. HADS is a brief and useful questionnaire to measure psychological disorders and symptom severity of anxiety disorders and depression. The HADS contains 14 items and consists of two subscales: anxiety and depression. Each item includes a four-point scale; higher scores indicate an elevated level of anxious and depressive symptomatology. Maximum score is 21 for anxiety and depression. Scores of 8 or more on either subscale were considered as psychological disorders and scores of 0–7 were defined as “normal” in the current study. The convergent validation of translated version of HADS questionnaire was examined in 167 Iranian adults using the correlation of each item with its hypothesized scale. Pearson’s correlation coefficients varied from 0.47 to 0.83 (*P* < 0.0001) for anxiety subscale and from 0.48 to 0.86 (P < 0.0001) for depression subscale, indicating that the questionnaire provides relatively valid measures of psychological health [19]. The Iranian validated version of General Health Questionnaire (GHQ) with 12-items was used to assess psychological distress [20]. GHQ-12 is a brief, simple, easy to-complete instrument for measuring current and primary mental health that asks the respondents whether they have experienced a particular symptom of psychological distress or a change in their behavior recently. Each item consists of a four-point scale (less than usual, no more than usual, rather more than usual, or much more than usual). We used the bimodal scoring style for this study. This gives scores ranging from 0 to 12. Higher scores indicate a greater degree of psychological distress. In the current study, psychological distress was defined as having the score of 4 or more [20]. The convergent validity of GHQ-12 was examined in 748 Iranian young people. Significant inverse correlation was seen between the GHQ-12 and global quality of life scores (r = − 0.56, *P* < 0.0001) [20].

### Assessment of other variables

Required information on other variables including age, sex, marital status, socioeconomic status (SES), smoking status, gestational and lactating status, chronic conditions (diabetes and colitis), and antidepressant and supplements (vitamins, minerals, calcium and iron) use was obtained from demographic and medical history questionnaires. SES score was computed as an index of socioeconomic status based on family size (≤4, > 4 people), education (academic and non-academic education), and acquisition (house ownership or not). Earlier studies have found that increased number of siblings was associated with decreased parental resources (such as time, energy, and money) [21], which can affect the socioeconomic status of family. The family size of four is considered as an acceptable family size in Iran society, therefore, we assumed that participants with family members of more than four have lower socioeconomic status than those with family members of less than four. Participants were given a score of 1 if they had family members of ≤4, were academically educated, or owned a house. Subjects were given a score of 0 if they had family members of > 4, or had non-academic education, or leasehold property. Then, total SES score was calculated by summing up the assigned scores (minimum SES score of 0 to maximum score of 3). Individuals with the score of 3 were considered as having high SES. Physical activity was assessed using the General Practice Physical Activity Questionnaire (GPPAQ) [22], and participants were classified into two categories: physically active (≥1 h/week) and physically inactive (< 1 h/week). Although this level of activity might seem low, but earlier publications have revealed that even 1 h per week of walking can reduce the risk of chronic conditions [23]. Anthropometric measures including weight, height, and waist circumference were assessed using a self-administered questionnaire. The validity of self-reported values of weight, height, and WC was examined in a pilot study on 200 participants from the same population. In the validation study, self-reported values of anthropometric indices were compared with measured values. The correlation coefficients for self-reported weight, height, and WC versus corresponding measured values were 0.95 (*P* < 0.001), 0.83 (*P* < 0.001), and 0.60 (*P* < 0.001), respectively. Body mass index was calculated by dividing weight (kg) to height (m^2^). The correlation coefficient for computed BMI from self-reported values, and the one from measured values was 0.70 (*P* < 0.001). Participants were categorized based on their BMI status into three groups: obese (BMI ≥ 30 kg/m^2^), overweight (25 ≥ BMI > 30 kg/m^2^) and normal (BMI < 25 kg/m^2^).

### Statistical analysis

Participants were classified based on quartiles of LCD score. To compare general characteristics of study participants across quartiles of LCD score, we used one-way ANOVA for continuous variables (including age, weight, BMI, and waist circumference) and Chi-square test for categorical variables. Mean dietary intakes of study participants across categories of LCD score were obtained by the using one-way ANOVA. The prevalence of depression, anxiety and GHQ across different categories of LCD score in the whole population were assessed by Chi-square. To investigate the relationship between adherence to LCD with depression, anxiety, and psychological distress, we used multivariable logistic regression analysis in different models. In these analyses, we controlled for age (year) and sex (male/female) in the first model. Additional adjustments were done for marital status (married/single/divorced/widow), socioeconomic status (high/moderate/low), smoking (yes/no), physical activity (less/more than1 h per week), presence of chronic diseases (yes/no), antidepressant (yes/no) and supplement use (yes/no); and pregnancy or lactation (yes/no). All confounding variables were chosen based on previous studies [24, 25]. In terms of chronic diseases, we controlled for diabetes and colitis, because these persons have been shown to be at higher risk of psychological disorders [26, 27]. Additional controlling was done for dietary fiber (g/d) and EPA plus DHA (g/d) intake. Finally, further adjustments for BMI (kg/m2) were done in the last model. In all these analyses, the first quartile of LCD score was considered as a reference. To assess the trend of odds ratios across increasing categories of LCD, we used the median score in each category as a continuous variable. Because the ORs estimated from logistic regression models are not valid estimators of the relative risk in cross-sectional studies when the outcome variables have higher prevalence than 10% in study population [27, 28], we used the formula suggested by Zhang and Yu [29] to correct the adjusted ORs obtained from logistic regression. All analyses were performed using Statistical Package for Social Sciences (SPSS Corp, version 18, Chicago, IL, USA). *P* values less than 0.05 were considered statistically significant.

## Results

Prevalence of depression, anxiety and psychological distress in the whole population were 28.0, 13.3 and 22.6%, respectively. General characteristics of study participants across quartiles of LCD score are shown in Table 1. Subjects in the highest quartile of LCD score had higher weight, BMI and waist circumference, were more likely to be male, obese, physically active, of high SES, university graduated than those in the lowest quartile. The prevalence of chronic conditions and smoking among them was higher than those in the bottom quartile. There was no significant difference in terms of other variables across quartiles of LCD score.

**Table 1:** General characteristics of study participants across the LCD score quartiles

	quartiles of LCD score				
	1(*n* = 887)	2(*n* = 816)	3(*n* = 803)	4(*n* = 856)	
LCD score range	< 10	10-16	16-23	> 23	P^b^
Age (year)a	36.1 ± 7.7	36.2 ± 7.8	36.3 ± 7.8	36.6 ± 8.1	0.57
Weight (kg)a	67.2 ± 12.4	67.9 ± 12.0	69.0 ± 13.6	70.5 ± 14.4	< 0.001
BMI (kg/m^2^)a	24.6 ± 3.7	24.8 ± 3.6	25.1 ± 3.8	25.2 ± 4.0	0.009
Waist circumference (cm)a	86.4 ± 10.9	87.0 ± 10.8	86.9 ± 11.4	88.9 ± 12.4	0.001
Overweight (%)	37	38.6	36.1	37	0.74
Obesity (%)	7.6	7.8	10.6	12.3	0.002
Doing exercise≥1 h/w (%)	9.8	11.2	14.9	16.9	< 0.001
Male (%)	38.7	36.8	41.7	49.6	< 0.001
Married (%)	79.9	81.9	84.2	80.9	0.38
High SES (%)	28.2	33.7	29.8	27.8	0.003
House owner (%)	65.4	68.9	70.8	71	0.07
University graduated (%)	64	68	59.5	56.1	< 0.001
Family size> 4 (%)	27.8	25.2	29	31.2	0.056
Chronic conditions (%)	2.8	3.7	4.9	7.2	< 0.001
Pregnant women (%)	2.6	1.9	1.8	1.7	0.75
Lactating women (%)	8.7	11.2	8.3	8.2	0.37
Current smoker (%)	10.6	12.1	17.3	15.4	< 0.001
Anti-depressant use (%)	5.3	4.4	6	6.5	0.26
Supplement use(%)	31.5	31	30.4	27.2	0.21

^a^ All values are Means±SD

^b^*P* values were obtained one-way ANOVA or χ2 test, where appropriate

**Fig. 2:**
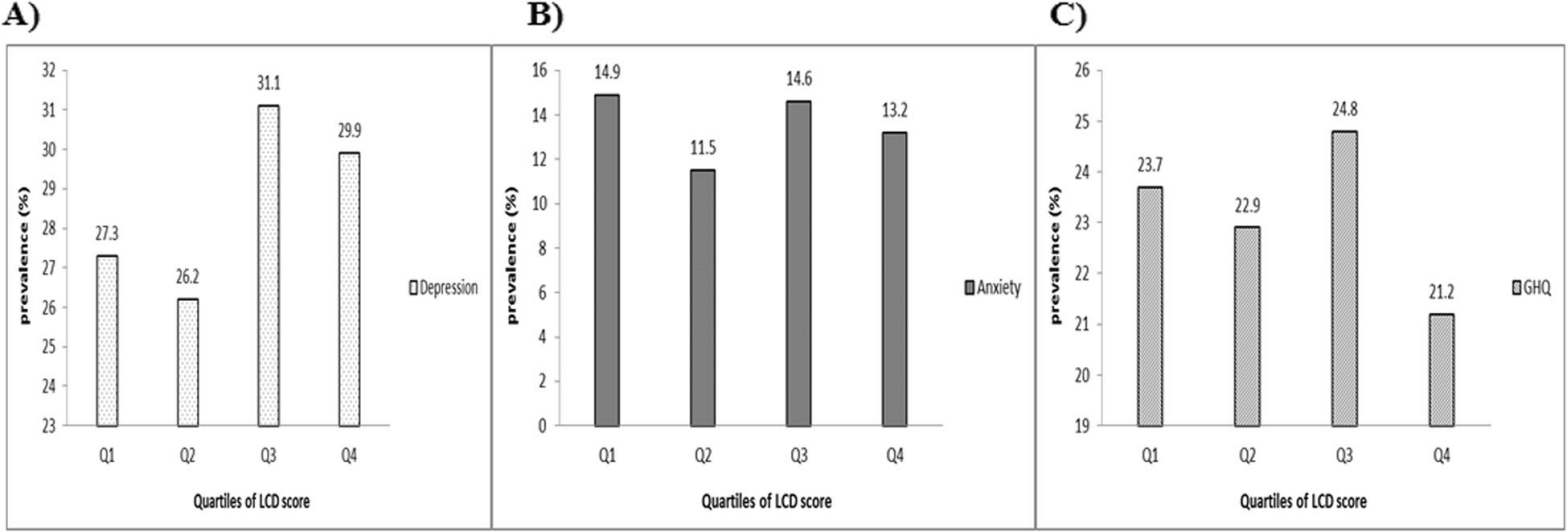
Prevalence of psychological disorders across different quartiles of LCD score in the whole population; **a** Depression; **b** Anxiety; **c** Psychological distress (GHQ)

Prevalence of depression across different categories of LCD score in the whole population is illustrated in Fig. 2. No significant differences were observed in the distribution of depression (*P* = 0.10), anxiety (*P* = 0.17) and psychological distress (*P* = 0.38) across different categories of LCD score.

Dietary intakes of participants are provided in Table 2. Compared to individuals without psychological disorders, participants with psychological problems had lower intakes of total fiber, EPA and DHA, vitamin B6, magnesium as well as fruits, vegetables, low fat dairy and higher intakes of refined grains. Participants in the top category of LCD score had higher daily intakes of energy, protein, fats, saturated fats, polyunsaturated fats, EPA and DHA, vitamin D, vitamin B_3_, vitamin B_6_, vitamin B_12_, vegetables, white meat, red meat, egg, legumes, nuts and soy; as well as high-fat dairy products than those in the bottom category. Subjects in the highest quartiles of LCD score had lower intakes of carbohydrate and total fiber, vitamin B_1_, folate, Iron, magnesium, fruits, refined grains, whole grains, sugar sweetened beverages and tea or coffee drinking than those in the bottom category of LCD score (*P* < 0.05 for all).

**Table 2:** Dietary intakes of study participants

	having psychological disorder			Quartiles of LCD score				
	No (n 2046)	Yes (n 1316)		1 (*n* = 887)	2 (*n* = 816)	3 (*n* = 803)	4 (*n* = 856)	
	Mean ± SD	Mean ± SD	P^a^	Mean ± SD	Mean ± SD	Mean ± SD	Mean ± SD	P^b^
Total energy (kcal/d)	2382.7 ± 823.1	2372.7 ± 830.7	0.73	2484.6 ± 861.1	2413.4 ± 809.1	2312.8 ± 806.3	2298.2 ± 810.2	< 0.001
Nutrients								
Carbohydrates (g/d)	293.7 ± 115.0	291.9 ± 119.1	0.67	362.8 ± 128.1	310.7 ± 105.0	267.4 ± 94.7	227.9 ± 85.6	< 0.001
Proteins (g/d)	88.7 ± 33.4	87.5 ± 33.3	0.35	80.6 ± 31.5	86.3 ± 30.8	87.4 ± 32.1	98.9 ± 36.1	< 0.001
Fats (g/d)	98.6 ± 36.9	98.4 ± 36.7	0.86	82.9 ± 29.5	95.8 ± 32.7	103.1 ± 37.1	113.4 ± 40.2	< 0.001
Saturated fats (g/d)	23.4 ± 9.2	23.0 ± 9.2	0.29	19.3 ± 7.3	22.7 ± 8.1	24.1 ± 9.0	27.1 ± 10.2	< 0.001
Polyunsaturated fats (g/d)	28.9 ± 11.7	29.0 ± 11.7	0.67	25.0 ± 9.6	28.1 ± 10.4	30.3 ± 12.3	32.6 ± 12.7	< 0.001
Total fiber (g/d)	22.8 ± 9.6	22.1 ± 9.6	0.04	25.3 ± 10.7	23.4 ± 9.1	21.3 ± 8.9	19.9 ± 8.6	< 0.001
EPA & DHA (g/d)	0.31 ± 0.3	0.29 ± 0.3	0.03	0.2 ± 0.2	0.2 ± 0.2	0.3 ± 0.2	0.4 ± 0.4	< 0.001
Vitamin D (mg/d)	36.9 ± 23.6	35.9 ± 22.7	0.20	28.3 ± 19.2	34.3 ± 19.5	39.9 ± 25.5	43.9 ± 25.1	< 0.001
Vitamin B_1_ (mg/d)	1.8 ± 0.9	1.8 ± 0.9	0.80	2.4 ± 1.2	1.9 ± 0.8	1.6 ± 0.6	1.4 ± 0.5	< 0.001
Vitamin B_3_ (mg/d)	24.9 ± 10.6	25.1 ± 10.8	0.58	27.0 ± 12.3	25.1 ± 10.5	23.3 ± 9.5	24.3 ± 9.8	< 0.001
Vitamin B_6_ (mg/d)	2.0 ± 0.7	1.9 ± 0.7	0.05	1.8 ± 0.7	1.9 ± 0.7	2.0 ± 0.7	2.2 ± 0.8	< 0.001
Folate (mg/d)	572.2 ± 235.2	573.3 ± 240.1	0.90	681.9 ± 281.6	599.2 ± 222.9	525.9 ± 192.8	477.9 ± 180.5	< 0.001
Vitamin B_12_ (μg/d)	3.0 ± 1.3	2.9 ± 1.3	0.09	2.2 ± 0.9	2.9 ± 1.1	3.1 ± 1.2	3.7 ± 1.5	< 0.001
Iron (mg/d)	17.5 ± 7.5	17.6 ± 7.6	0.56	20.3 ± 9.0	18.07 ± 7.3	16.20 ± 6.4	15.45 ± 6.02	< 0.001
Magnesium (mg/d)	330.4 ± 120.9	322.1 ± 118.2	0.05	333.4 ± 123.1	339.1 ± 121.5	320.1 ± 117.8	315.8 ± 115.6	< 0.001
Food groups								
Fruits (g/d)	332.9 ± 246.8	289.5 ± 233.8	< 0.001	395.4 ± 313.0	342.77 ± 221.6	293.7 ± 203.8	229.0 ± 170.2	< 0.001
Vegetables (g/d)	242.7 ± 128.3	230.0 ± 135.1	0.006	210.3 ± 119.2	239.0 ± 123.3	248.6 ± 132.6	254.6 ± 144.0	< 0.001
White meat (g/d)	65.1 ± 48.8	63.2 ± 49.7	0.26	38.0 ± 25.9	54.3 ± 33.4	67.1 ± 44.5	98.7 ± 62.3	< 0.001
Red meat (g/d)	78.2 ± 48.2	79.6 ± 48.9	0.42	52.5 ± 31.1	71.2 ± 37.2	84.7 ± 44.7	108.0 ± 58.3	< 0.001
Egg (g/d)	25.0 ± 19.8	25.0 ± 21.3	0.95	18.9 ± 15.8	22.7 ± 71.2	27.5 ± 20.1	31.2 ± 25.0	< 0.001
Legumes, nuts and soy (g/d)	57.1 ± 38.8	58.1 ± 41.8	0.49	46.5 ± 31.9	56.0 ± 39.9	60.6 ± 40.4	67.6 ± 44.2	< 0.001
Low fat dairy (g/d)	344.6 ± 276.1	313.6 ± 259.4	0.001	277.9 ± 213.5	360.1 ± 282.4	349.6 ± 279.3	346.5 ± 293.2	< 0.001
High fat dairy (g/d)	15.0 ± 18.6	14.8 ± 18.5	0.77	13.5 ± 17.6	14.5 ± 18.7	15.8 ± 18.3	15.9 ± 19.0	0.018
Refined grains (g/d)	384.1 ± 211.6	400.8 ± 221.4	0.03	495.2 ± 277.8	407.6 ± 202.7	350.4 ± 158.6	303.8 ± 139.0	< 0.001
Whole grains (g/d)	42.9 ± 74.8	41.4 ± 80.7	0.58	58.8 ± 105.6	51.3 ± 83.0	34.2 ± 56.9	24.3 ± 38.8	< 0.001
Sugar sweetened beverages and sweets (g/d)	52.1 ± 53.0	55.0 ± 58.0	0.14	63.3 ± 66.0	52.55 ± 54.6	51.4 ± 50.2	45.1 ± 45.1	< 0.001
Tea or coffee drinking (mg/d)	371.4 ± 281.3	388.4 ± 305.6	0.09	397.9 ± 278.5	387.3 ± 280.0	377.8 ± 288.6	348.9 ± 313.9	0.04

^a^Obtained by Independent sample t-test

^b^Obtained by ANOVA

Crude and multivariable-adjusted odds ratios and 95% CIs for psychological disorders across categories of LCD score are indicated in Table 3. No significant associations were seen between LCD score and prevalence of depression in crude model (OR: 1.09; 95% CI: 0.94, 1.26). When we considered several potential confounders, this association did not change (OR: 1.15; 95% CI: 0.93, 1.39). The same association were reached between adherence to the LCD diet and odds of anxiety and psychological distress (OR: 0.82; 95% CI: 0.59, 1.14 and OR: 0.92; 95% CI: 0.72, 1.16, respectively). The associations did not change when the analyses were done stratified by gender or BMI status.

**Table 3:** Multivariable-adjusted ratios for psychological disorders across quartiles low-carbohydrate diet (LCD) score

	Quartiles of LCD score				
	1 (*n* = 887)	2 (*n* = 816)	3 (*n* = 803)	4 (*n* = 856)	P trend
	OR	OR	OR	OR	
LCD score range	< 10	10-16	16-23	> 23	
Depression					
Crude	1.00	0.95(0.81–1.12)	1.14(0.98–1.31)	1.09(0.94–1.26)	0.06
Model I	1.00	0.93(0.78–1.10)	1.10(0.93–1.29)	1.12(0.95–1.31)	0.05
Model II	1.00	1.00(0.82–1.20)	1.12(0.93–1.33)	1.13(0.94–1.33)	0.11
Model III	1.00	1.01(0.83–1.21)	1.13(0.94–1.36)	1.18(0.96–1.41)	0.06
Model VI	1.00	1.00(0.82–1.21)	1.13(0.92–1.36)	1.15(0.93–1.39)	0.11
Anxiety					
Crude	1.00	0.77(0.59–0.99)	0.97(0.77–1.23)	0.88(0.69–1.11)	0.64
Model I	1.00	0.72(0.55–.094)	0.93(0.72–1.20)	0.90(0.69–1.15)	0.79
Model II	1.00	0.73(0.54–0.98)	0.87(0.65–1.16)	0.87(0.65–1.15)	0.52
Model III	1.00	0.71(0.52–0.96)	0.83(0.61–1.12)	0.80(0.59–1.11)	0.29
Model VI	1.00	0.70(0.51–0.96)	0.85(0.62–1.15)	0.82(0.59–1.14)	0.40
Psychological distress					
Crude	1.00	0.96(0.81–1.14)	1.04(0.87–1.23)	0.89(0.74–1.07)	0.38
Model I	1.00	0.93(0.77–1.12)	1.05(0.87–1.25)	0.93(0.76–1.12)	0.78
Model II	1.00	1.03(0.84–1.26)	1.05(0.85–1.28)	0.98(0.79–1.19)	0.88
Model III	1.00	1.01(0.82–1.23)	1.01(0.80–1.24)	0.92(0.73–1.15)	0.51
Model VI	1.00	1.01(0.81–1.24)	1.04(0.82–1.28)	0.92(0.72–1.16)	0.59

Model I: adjusted for age, sex

Model II: adjusted for Model 1 plus marital status (category), socioeconomic status (category), smoking (category), physical activity (category), chronic disease (category), antidepressant use (category), supplement use (category)

Model III: all variables in Model II plus dietary fiber, EPA plus DHA

Model VI: additionally adjusted for BMI

## Discussion

This cross-sectional study in a large group of Iranian population revealed no significant association between adherence to the LCD and prevalence of psychological disorders. To the best of our knowledge, this is the first observational study examining the association between adherence to the LCD and risk of psychological disorders.

Pervious observational studies had mostly focused on individual intakes of dietary macronutrients instead of whole dietary macronutrients. In line with ours, a cross-sectional study in Japanese men revealed that neither carbohydrate nor fat intake was associated with depressive symptoms [4]. In contrast, Pellegrin et al. reported that high carbohydrate intake was related to lower anxiety and depression [6]. Some others also suggested an inverse association between high carbohydrate and high protein intakes and depression [30]. Prescribing a low-carbohydrate diet resulted in less confusion than American Dietetic Association’s diet [7]. Consumption of a low carbohydrate diet along with high protein have been suggested for psychological health of obese women [11]. In addition to quantity, some studies have investigated the association between carbohydrate quality and risk of psychological disorders. For instance, high Glycemic Index (GI) diets was associated with greater risk of depression and psychological disorders [9, 10]. Data on fat intake and depression are limited to type of fats. In a prospective study on Spanish population, Sanchez-Villegas et al. showed an inverse relationship between dietary tarns unsaturated fatty acids intake and risk of depression [31]. Naturally, considering the whole dietary macronutrients together in relation to psychological health is totally different from the studies done on single macronutrients. We failed to find any relation between adherence to the LCD and these disorders. However, it must be kept in mind that we could not consider type of carbohydrates, fats and proteins. Specific fats and proteins might have different effects on psychological health. This is also the case for dietary carbohydrates, where the association between high vs. low GI diets and psychological health might be different [10]. Despite adverse effects of sugar and simple carbohydrates on anxiety and depression, low intakes of carbohydrates and hypoglycemia were shown to lower levels of serotonin and increase psychological disorders [10, 30]. Overall, it seems that further studies are required to examine the relationship between different types of macronutrients together in relation to psychological health.

There were some limitations in this cross-sectional study that should be considered in interpretation of our findings. Due to the cross-sectional design of this study, causal relations cannot be inferred. However, it must be kept in mind that appropriate analysis of cross-sectional data represent a valuable initial step in identifying relationships between diet and disease. We used self-administered questionnaires for assessment of both exposure and outcomes. Therefore, misclassification of study participants in terms of both exposure and outcome cannot be excluded. This would result in some sort of bias which would move the odds ratios toward null associations. Moreover, there were some inevitable measurement errors in assessment of anthropometric indices, diet and physical activity. However, using validated questionnaire and adjusted ORs may cover some of these errors. In addition, the study population of SEPAHAN were all staffs of the university. Therefore, the findings cannot be easily extrapolated to other populations. Although we consider a wide range of confounding factor, the effect of residual confounding cannot be avoided.

## Conclusion

In conclusion, we failed to find any significant association between adherence to the low carbohydrate diet (LCD) and odds of psychological disorders in this cross-sectional study. Despite earlier evidence that support the association between individual macronutrients and mental problems, it seems that combination of high fat and protein intakes along with lower intake of carbohydrates as components of LCD score are not relates to psychological disorders in our study population. Further longitudinal studies, focusing on types of macronutrients, are required to clarify this association.

## Data Availability

Not applicable.

## Abbreviations

BMI: Body Mass Index
DHA: Docosahexaenoic Acid
DS-FFQ: Dish-based Semi-quantitative Food Frequency Questionnaire
EPA: Eicosapentaenoic Acid
GHQ: General Health Questionnaire
GI: Glycemic Index
GPPAQ: General Practice Physical Activity Questionnaire
HADS: Hospital Anxiety and Depression Scale
LCD: Low Carbohydrate Diet
SEPAHAN: Study on the Epidemiology of Psychological, Alimentary Health and Nutrition
SES: Socioeconomic Status
WC: Waist Circumference
